# Alternative hypotheses to explain why biodiversity-ecosystem functioning relationships are concave-up in some natural ecosystems but concave-down in manipulative experiments

**DOI:** 10.1038/srep05427

**Published:** 2014-06-25

**Authors:** Camilo Mora, Roberto Danovaro, Michel Loreau

**Affiliations:** 1 Department of Geography, University of Hawaii, Honolulu, Hawaii, USA; 2 Department of Life and Environmental Sciences, Polytechnic University of Marche, Ancona, Italy; 3 Stazione Zoologica Anton Dohrn, Villa Comunale, 80121, Naples, Italy; 4 Centre for Biodiversity Theory and Modelling, Station d'Ecologie Expérimentale du CNRS, 09200 Moulis, France

## Abstract

Recent studies of the relationship between biodiversity and functioning in marine ecosystems have yielded non-saturating patterns that contrast sharply with the results of experimental studies, where ecosystem functioning rapidly saturates with increases in biodiversity. Here we provide a simple theoretical framework of three alternative hypotheses that, individually or combined, are likely to explain this contrast: *i*) the use of functional richness instead of species richness, *ii*) an increased production efficiency of species in producing biomass when more ecological interactions are present, and *iii*) the fact that communities are likely assembled in an ordered succession of species from low to high ecological efficiency. Our results provide theoretical support for concave-up biodiversity-ecosystem functioning relationships in natural ecosystems and confirm that the loss of species can have substantially larger effects on the functioning of natural ecosystems than anticipated from controlled manipulative experiments.

Ongoing losses of biodiversity have raised concerns over its consequences for the functioning of ecosystems and subsequent shortfalls in the supply of ecosystem goods and services to humanity^1^,^2^. As a result, there has been great scientific interest in assessing the relationship between biodiversity and ecosystem functioning (BEF hereafter)^2^,^3^,^4^,^5^,^6^,^7^,^8^,^9^. Given the complexity of natural ecosystems, the broad spatial scales they cover and the large number of environmental variables that can influence the results, research into BEF relationships has been, so far, overwhelmingly based on experimental approaches^10^,^11^. Experimental studies have consistently shown a saturating (concave-down) relationship between ecosystem functioning (e.g. standing stock and productivity) and biodiversity (e.g. species and functional richness), with slopes on log-log scales ranging from 0.15 to 0.32 [Fig. 1a^7^] (in log-log scale, slopes smaller than 1 represent concave-down trends whereas slopes larger than 1 are concave-up). However, two recent large-scale observational studies on marine systems, one on deep-sea nematodes^12^ and the other on coral reef fishes^13^, have yielded steeper, non-saturating trends with increased biodiversity, with slopes on log-log scales ranging from 1.1 to 8.4 (Table 1, Fig. 1b). Further, a meta-analysis conducted on different marine systems has showed that ecosystems processes related to stability and water quality decreased exponentially with declining diversity^14^.

An emerging question from these studies on natural ecosystem raises is why their results differ so strongly from the pattern commonly found in experimental manipulative experiments^2^,^15^. Existing theory for single-trophic-level in competitive systems consistently predicts saturating, concave-down BEF relationships; the only exception occurs when species interactions turn from competitive to mutualistic on average^16^,^17^. Since communities structured entirely or predominantly by mutualistic interactions between species should be an exception, the traditional hypothesis to explain discrepancies between observational and experimental studies is the presence of confounding environmental factors^2^,^3^,^18^. But additional statistical analyses conducted on deep-sea nematodes and coral reef fishes revealed that the concave-up BEF relationships found in the above studies were still highly significant even after controlling for the effects of potential confounding environmental factors such as depth, food supply and other variables, either independently or simultaneously^12^,^13^. Thus, confounding environmental factors do not appear to explain the discrepancy between these marine observational investigations and most experimental studies, leaving the question unanswered so far.

Concave-up and concave-down BEF relationships have very different ecological implications, especially in relation to the effects of biodiversity loss. In the case of a concave-down pattern, theoretically it would be acceptable to lose a few species without major consequences for ecosystem functioning. Conversely, in the case of a concave-up pattern, the loss of species will cause exponential declines in ecosystem functioning. These implications are an additional motivation to explain why marine observational studies and previous experimental work yield such contrasting responses. Explaining why these differences occur has been recently highlighted as an important research need in the BEF research field^2^. We caution that our interest is not to indicate the commonality of concave-up relationship in nature, but rather elaborate hypotheses that may conciliate contrasting shapes for BEF relationships.

In this study, we provide a set of three hypotheses that are likely to explain the different slopes of BEF relationship in marine observational studies and manipulative experiments: *i*) the use of functional richness instead of species richness, *ii*) an increased production efficiency of species at producing biomass in the presence of ecological interactions, and *iii*) the fact that communities are likely assembled in an ordered succession of species from low to high production efficiency. Although discerning among these hypotheses will be challenging, they all provide theoretical rational for the contrast in the slopes of BEF relationships between natural ecosystems and experiments and suggest that the loss of species in the real world may have larger consequences than those anticipated from manipulative experimental studies.

## Concave-up BEF relationships in marine ecosystems

**Figure 1:**
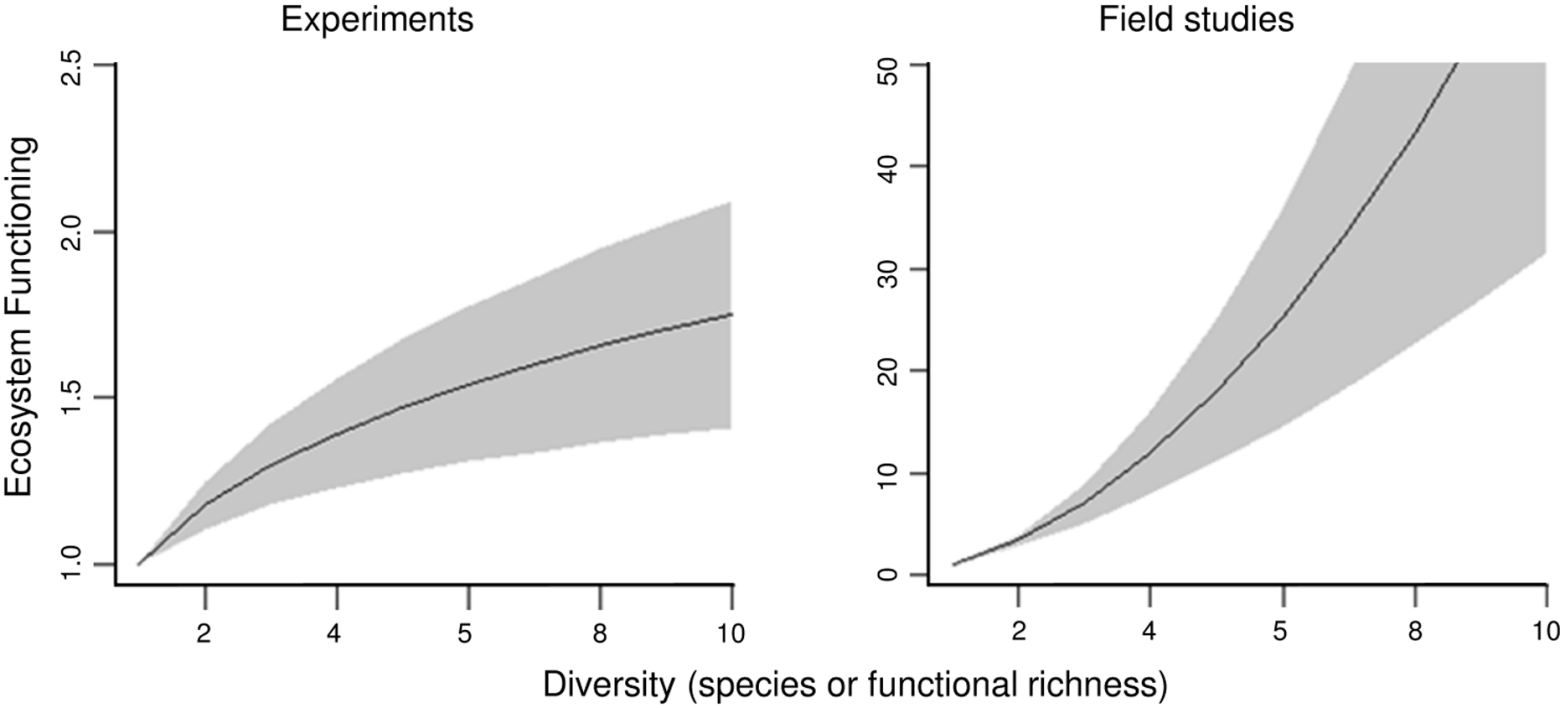
Experimental (a) and natural (b) ecosystems relationships between biodiversity and ecosystem functioning. Plot **a** shows the 95% confidence limits of the log-log slopes yielded by 111 experiments analyzed^7^. Plot **b** show the range of slopes yielded by field studies on coral reef fishes and deep-sea nematodes (Table 1).

BEF relationships have been assessed in four independent regions [i.e., Caribbean, Eastern Pacific, Indo Pacific and Indian Ocean] for fishes on coral reefs^13^, and in three ocean basins [i.e., Pacific, Atlantic and Mediterranean] for nematodes in the deep-sea^12^. We focus on these specific studies as in our knowledge they are the only examples showing clear concave-up BEF relationships and their data were readily available to the authors for standardized comparison of model parameters (Table 1). For both ecosystems, biodiversity metrics included species and functional richness while the metrics for ecosystem functioning were standing stock and secondary production. The BEF relationship in these natural ecosystems revealed two important characteristics. First, functional diversity yielded steeper relationships than did species richness (Table 1). Second, regardless of the ecosystem, region or use of species or functional richness, the log-log slopes of the biodiversity-ecosystem functioning relationships in these natural ecosystems were markedly steeper than those documented in experimental studies; that is 1.1 to 8.4 (n = 7 independent regions encompassing 2 ecosystems) versus 0.15 to 0.32 in experimental studies [n = 111 experiments^7^](Table 1, Fig. 1). We acknowledge that, despite the hundreds of sites examined, the number of BEF relationships for natural ecosystems is much smaller than those available from experiments. This is a practical limitation due to the high cost and challenges of the transnational organization required to collect detailed data over large scales. Thus, the generality of concave-up BEF relationships in other natural ecosystems is currently unknown. The two available case studies, however, strongly suggest some generality, especially among other closely related marine ecosystems, as the concave-up BEF relationships were common in systems separated not only spatially (i.e., shallow vs. deep and over different ocean domains), but also phylogenetically (i.e., vertebrate vs invertebrate). Irrespective of the generality of concave-up BEF relationships in other ecosystems, explaining this pattern is worth in its own right for key ecosystems like coral reefs and the deep-sea.

## Why are BEF relationships steeper with functional richness than with species richness?

**Figure 2:**
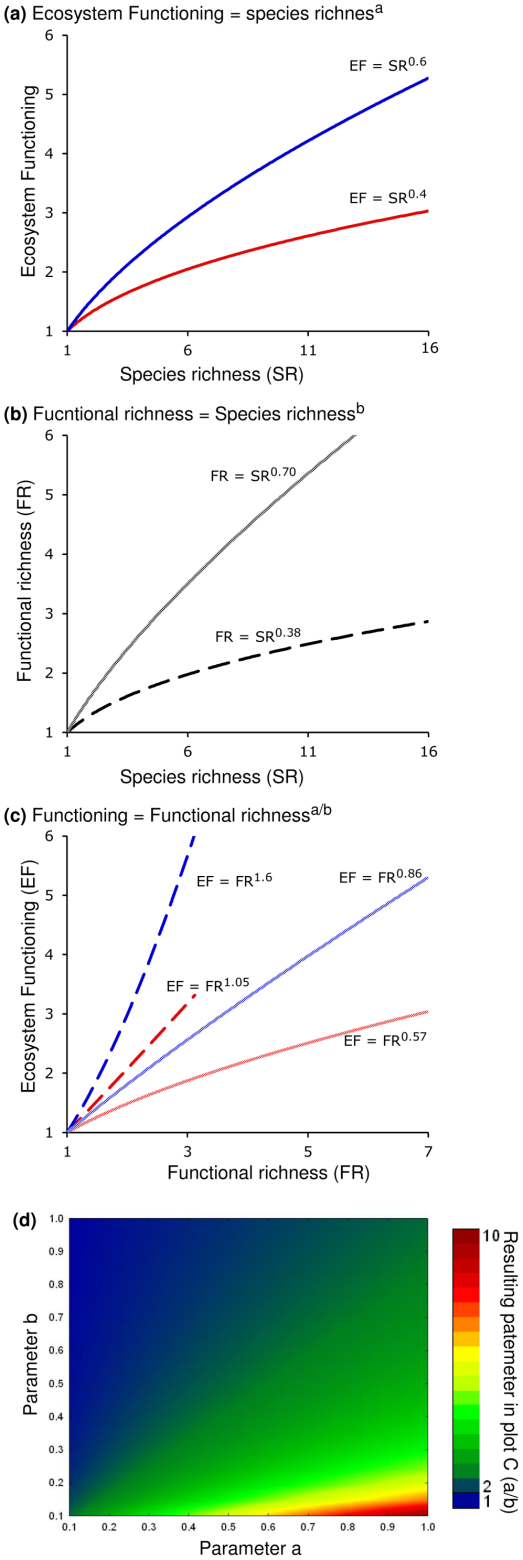
Effect of functional richness and species richness on biodiversity-ecosystem functioning relationships. These plots are intended to illustrate how concave-down biodiversity-ecosystem functioning using species richness (Plot **a**), depending on the relationship between species richness and functional richness (Plot **b**), can range from concave-down to concave-up with the use of functional richness (Plot **c**). Plot (**c**) show the combinations of all examples in plot A and plot B, which are coded by color and line type. Plot (**d**) shows the resulting power parameter of the functional richness-ecosystem functioning relationship depending on the power parameters of the species richness-ecosystem relationship (i.e., power parameter **a** in plot (**a**), equation 1) and the power parameter of the functional richness-species richness relationship (i.e., power parameter **b** in plot B, equation 3).

The relationship between ecosystem functioning (***EF***) and species richness (***SR***) is generally well fitted by a power model of the form: 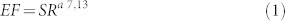
(See examples in Fig. 2a).

It is known that other models such as Michaelis-Menten and hyperbolic functions also offer good fit for BEF relationships although the differences are minimal and their use prevents combining other factors in a common mathematical framework. Cardinale et al.^11^ showed that the best fit for BEF relationships was provided by a Michaelis-Menten function but the difference was not considerable when compared to the power model. Additionally, the Michaelis-Menten function cannot be used for concave-up relationships, which is a serious limitation for comparing different types of relationships, especially those emerging from observational marine studies. Therefore, we chose the power model as it provides a simple common ground for comparison of concave-down and concave-up relationships. We emphasize that the power model is used simply as a mathematical way to represent general trends in ecological patterns; no assumption is made about its biological meaning.

In turn, the relationship between functional richness (***FR***) and species richness (***SR***) most often follows a concave-down pattern, which can also be described by a power model of the form: 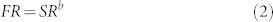
(See examples in Fig. 2b).

Substituting equation 2 into equation 1 gives the following relationship between ecosystem functioning and functional richness: 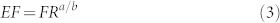
(see Fig. 2c).

The power exponent ***b*** is expected to vary considerably among studies depending on the number of functional groups considered relative to the number of species. If the number of functional groups is equal to the number of species then ***b*** = 1, in which case a linear relationship with a slope of 1 will define the relation between functional richness, ***FR***, and species richness, ***SR***. In general, however, functional richness is measured as the total number of functional traits of the species in the ecosystem and typically there are more species than traits. Therefore, parameter ***b*** is expected to be generally smaller than 1. The only exception is when each species have multiple functional roles and the number of functions exceeds the number of species, which is possible in principle but uncommon. As a result, the power parameter of the functional richness-ecosystem functioning relationship (equation 3) is divided by a number smaller than one, which yields a steeper slope than when species richness is used (equation 1) (see examples in Fig. 2).

This result of methodological constraints (i.e., more species than functional traits are generally measured) provides a parsimonious explanation that has not been made explicit so far for why BEF relationships are generally steeper with the use of functional richness than with the use of species richness. As a consequence, classifications of species in functional groups can have considerable effects on the shape of BEF relationships that use functional diversity. As an example, Acanthurid fish species in coral reefs are commonly classified as a single herbivorous functional group. However, detailed analysis of their diet and habitat use suggests that Acanthurid species specialize in the grazing of different species of algae at different places^19^. Thus, this single functional group is, in fact, an aggregate of a wide range of species that play different roles in coral reef functioning; how these species are aggregated in functional groups will considerably influence the slope BEF relationship. This situation is probably common in many other species and functional groups. This simple characteristic of how we measure biodiversity is also important because it generates concave-up BEF relationships in cases where parameter ***a*** is larger than parameter ***b***. In such cases, the power coefficient of the functional richness-ecosystem functioning relationship (equation 3) is larger than one, i.e., the relationship is concave-up (see examples in Fig. 2). There is no reason to suspect that parameters ***a*** and ***b*** are related and thus no argument for why one should be larger or smaller than the other (they are simple intrinsic attributes of the system). However, the potential for functional richness, instead of species richness, to generate concave-up BEF relationships, is insufficient to reconcile the results of experimental studies and those of marine observational studies because even with the use of species richness, BEF relationships in these natural ecosystems are still concave-up and significantly steeper than in manipulative experimental studies (Table 1).

## Why are BEF relationships steeper in marine observational studies than in experimental studies?

### The dual effect of ecological interactions on species' population size and production efficiency

In both experimental settings and natural ecosystems, ecological interactions among species are expected to affect ecosystem functioning in two different ways: *i)* by changing the population size of the various species, and *ii)* by changing their production efficiency, defined here broadly as the capacity of a species to produce biomass, through adaptive changes.

Traditional models in theoretical ecology have considered only the effects of species interactions on population size and have ignored their potential effects on species' adaptive changes. For instance, Lotka–Volterra models assume constant carrying capacities and interaction coefficients, but omit potential changes in species' production efficiency. These models predict that competitive interactions generate concave-down BEF relationships whereas mutualistic interactions generate concave-up relationships^15^,^16^. However, ecological interactions (e.g., competition, predation, etc) can also induce considerable adaptive changes^20^, which in turn may affect ecosystem functioning substantially. Adaptive responses to ecological interactions can range from short-term behavioral responses, to medium-term physiological and developmental phenotypic plasticity, to long-term evolutionary changes^21^. As an example, competition and predation can reduce individual body mass because of investment of energy to defend territories or to cover larger foraging areas or because of reduction in foraging time and places to avoid predators. Ecological interactions can also lead to niche shift over ecological time or character displacement over evolutionary time. These adaptive changes often result in increasing specialization-or more efficient use of available resources- in the presence of interacting species. For instance, studies of dietary and habitat specialization, potentially caused by intense competition, have shown that fishes increase their growth when feeding upon their preferred prey^22^ or when they reside on specific habitats^23^. Predation and competition are also known to trigger faster somatic growth to gain competitive advantage or escape size-dependent predation^24^,^25^,^26^,^27^,^28^; this will rapidly add to both production and standing stock of the community since prey body size will be larger and prey will growth faster to escape early mortality. Predation and competition can also cause early sexual maturation, leading to greater offspring production to compensate for induced mortality^24^. Several recent studies have showed that niche shifts contribute to the positive BEF relationships in both plants^29^ and insect pollinators^30^ even in small-scale experimental settings. Differential exploitation by predators creates a new niche axis that allows niche differentiation and hence complementarity between species^17^,^31^,^32^. Another ecological interaction, which is often underestimated, is facilitation, which can favor population and body size growth in at least one of the interacting species while causing harm to neither^12^,^33^. In short, by influencing species' production efficiency, ecological interactions have the potential to greatly influence ecosystem functioning.

To account for the dual effect of species interactions on ecosystem functioning through changes in the population size and production efficiency of the various species when species richness varies, we make three simplifying assumptions. First, ecosystem functioning (*EF*), as measured by some aggregate ecosystem properties such as total biomass, is the product of three terms:

- the average contribution of each species to ecosystem functioning in the absence of species interactions (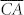),
- the net effect of species interactions on the contribution of each species (*NE*), and
- the number of species (*SR*).

**Figure 3:**
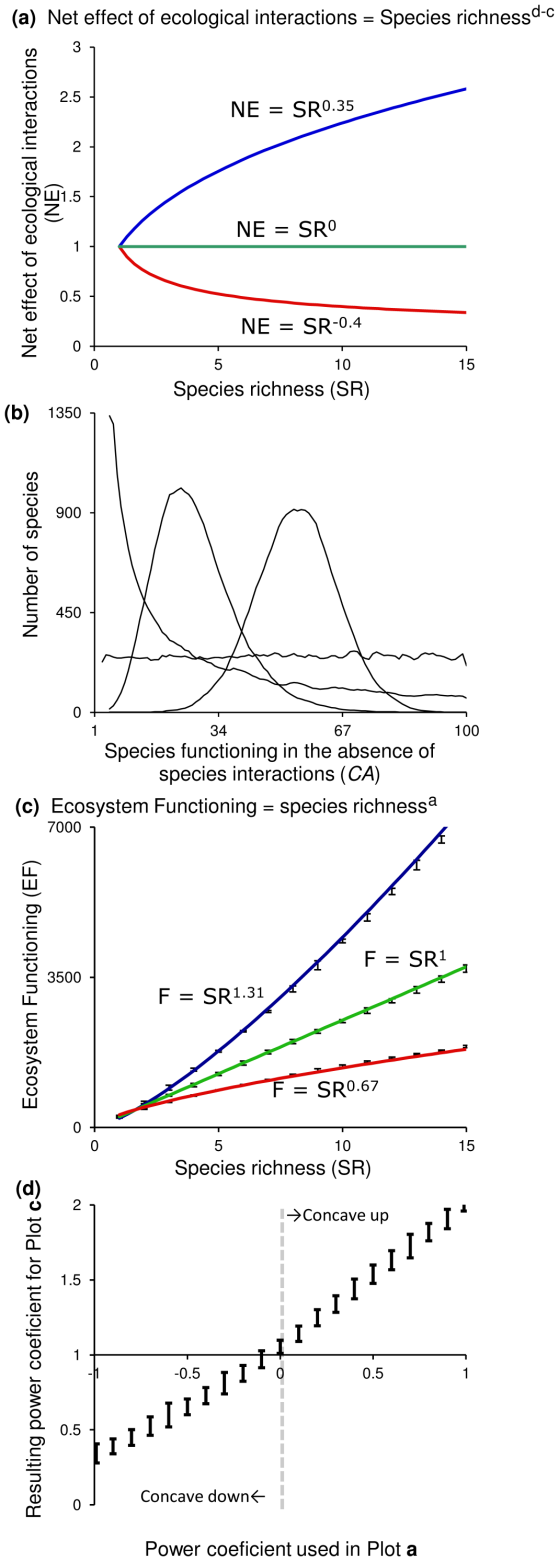
Effect of ecological interactions on biodiversity-ecosystem functioning relationships. Plot (**a**) shows examples of negative (i.e., ***d-c***<0, red line), neutral (i.e., ***d-c*** = 0, green line) and positive (i.e., ***d-c***>0, blue line) net effects of ecological interactions on population size and production efficiency (from equation 8). Plot (**b**) shows a range of hypothetical species contributions to ecosystem functioning in the absence of ecological interactions. Plot (**c**) shows the biodiversity-ecosystem functioning relationship for species randomly selected from plot (**b**) whose ultimate functioning is modified by the net effect of ecological interactions in plot (**a**). Plot (**d**) shows the power parameter of biodiversity-ecosystem functioning relationships depending on the net effect of ecological interactions (i.e., value of ***d-c***). Note than whenever the net effect of ecological interactions is larger than zero, biodiversity-ecosystem functioning relationships are concave-up. Vertical lines in plots (**c**–**d**) indicate confidence intervals from using the different frequency distributions in plot (**b**).

Second, the net effect of species interactions on the contribution of each species (*NE*) is itself the product of two terms, one due to changes in population size (*PS*) and another due to changes in production efficiency (*PE*). Third, the effects of ecological interactions on population size and on production efficiency are power functions of species richness (e.g. Fig 3a). These assumptions yield the following equations: 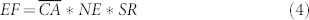

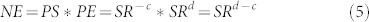
and hence 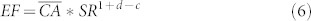
In these equations, ***c*** is the power coefficient that captures the effect of species interactions on ecosystem functioning through changes in population sizes (where ***c*** measures the strength of the reduction in population size generated by competition or predation), and ***d*** is the power coefficient that captures the effect of species interactions on ecosystem functioning through changes in production efficiency. In turn, ***d-c*** determines the net effect of species interactions on the contribution of each species to ecosystem functioning (equation 5), and the sum 1+*d*-*c* determines the total effect of species richness on ecosystem functioning.

**Figure 4:**
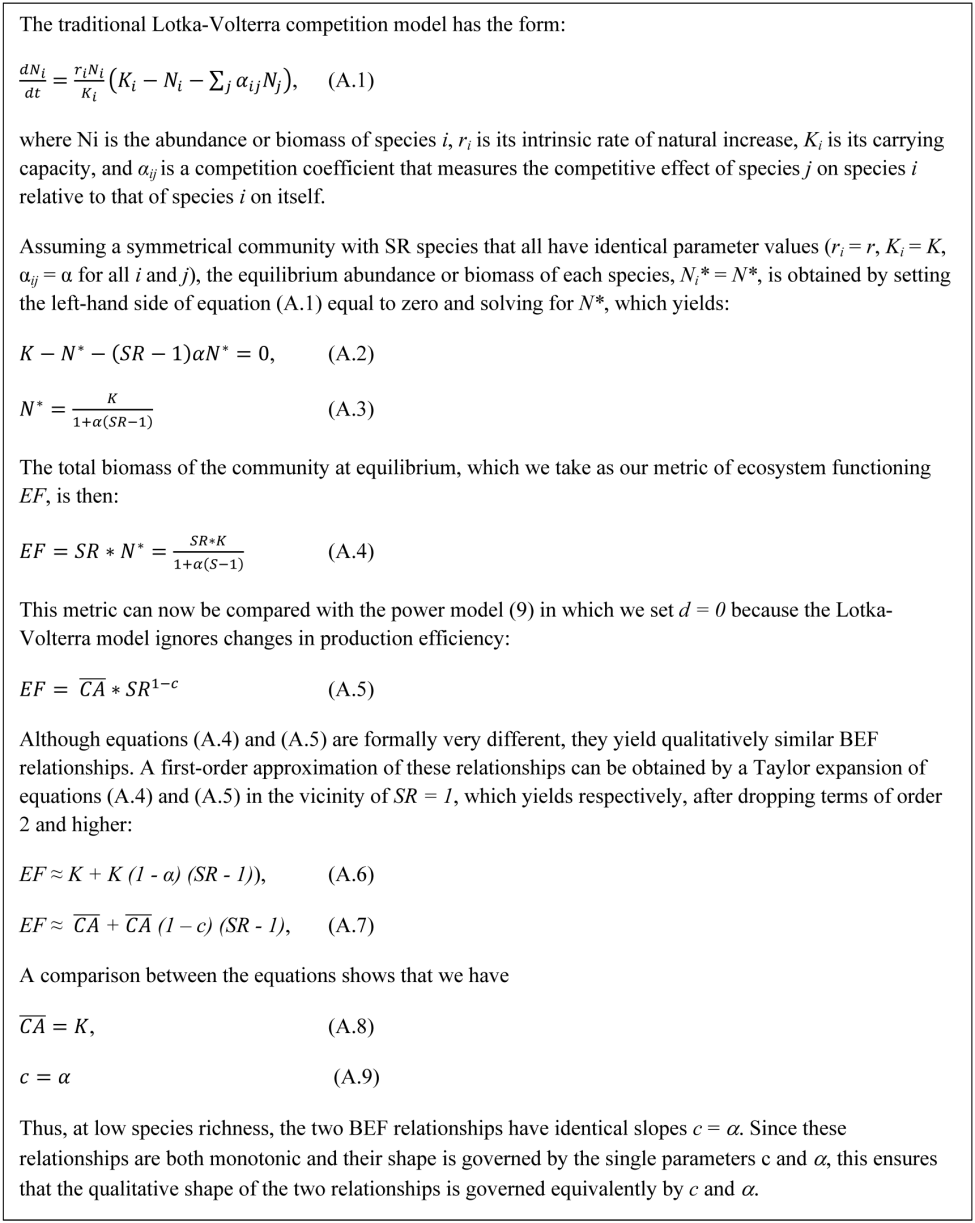
Lotka-Volterra competition models including changes in population size. Relationship between the competition coefficient in the Lotka-Volterra competition model and the power coefficient c (equations 8 and 9), which captures the effect of species interactions on ecosystem functioning through changes in population sizes.

In a symmetrical community obeying Lotka–Volterra dynamics, there is a simple approximate relationship between the interspecific competition coefficient, ***α***, and the power coefficient, ***c***, that measures the strength of the reduction in population size generated by species interactions at low species richness (see demonstration in Fig. 4): 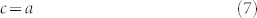
Although equation (7) is valid only at low species richness, since the BEF relationships predicted by the power and Lotka–Volterra models are both monotonic and their shape is governed by the single parameters *c* and *α*, equation (7) ensures that the qualitative shape of the BEF relationships is governed equivalently by *c* and *α*. Thus, ecosystem functioning (equation 6) is unaffected by species richness through changes in population sizes when interspecific competition is maximum (*c* = *α* = 1; note that *α* can technically be larger than 1 but then no stable coexistence is possible); it increases linearly when interspecific competition is minimum (*c* = *α* = 0); and it yields a concave-down BEF relationship when interspecific competition is intermediate (0 < *c*, *α* < 1), in agreement with previous theory^16^,^18^. In traditional Lotka-Volterra models, the only way the BEF relationship can be concave-up is when changes in population size are driven by mutualistic interactions (*c*, *α* < 0).

But as noted earlier, the Lotka-Volterra model ignores changes in species' production efficiency. If ecological interactions among species increase specialization and potential of facilitation, leading to increased production efficiency (which is likely as we illustrated with multiple examples earlier), the power parameter ***d*** is positive; ***d*** could also be negative in cases where interactions reduce production efficiency. The potential for ***d*** ranging from negative to positive broadens the spectrum of possible BEF relationships since even in purely competitive communities BEF relationships can be concave-up provided species respond to ecological interactions through production efficiency positively and more strongly than through population size (***d*** > **c**). Under these conditions, adding more species will increase ecosystem functioning (equation 5) (i.e., the exponent 1+*d*-*c* > 1 in equation 6). Alternative scenarios of this framework are presented in Fig. 3.

The effect of ecological interactions on ecosystem functioning through changes in species' production efficiency is an important theoretical result as prior theory considered mainly the role of competitive interactions on population sizes and always predicted concave-down BEF relationships. Expanding traditional theory of communities obeying Lotka–Volterra dynamics (to include adaptive changes in species' production efficiency) shows that concave-down relationships in competitive communities can be changed into concave-up relationships. This occurs when the effect of ecological interactions on species' production efficiency is positive and larger than their effect on population size. This suggests that experiments can easily fail to reveal the positive role of ecological interactions on species' production efficiency, as competition, instead of specialization, is more likely to prevail in experimental settings. In other words, the “ghost of competition past” may not be evident in experiments as much as it is in natural systems. That is, when species are put together in a contained artificial experimental setup they are forced to compete or interact, which may lead to greater energy loss than under field conditions where specialization may have already occurred. Gravel et al.^34^ showed, for instance, that the BEF relationship changes as a result of niche evolution. Likewise, Reich et al.^35^ showed that over time the BEF relationship gets steeper, thus supporting the hypothesis of a greater specialization and a decrease in the energetic costs associated with competitive interactions early on in the experiments. Experiments are also carried out using small number of species, which reduces the spectrum and strength of ecological interactions likely to occur on natural systems. In all the studies reviewed in Covich et al.^10^, for instance, the highest number of species considered was 22, 75% of the studies considered less than 7 species. It is interesting to notice that if the data reported here were limited to the first 22 species encountered, the exponential relationships reported would not be evident nor significant.

The role of ecological interactions on species' production efficiency provides a parsimonious explanation for concave-up BEF relationships in diverse ecosystems like coral reefs or the deep-sea, where ecological interactions are likely to be strong and numerous and may have already led to resource specialization. We acknowledge that providing empirical support for this hypothesis will be challenging as measuring changes in species' production efficiency will require detailed measurements of individual-level responses, perhaps over evolutionary scales. Recent experiments have shown that evolutionary and long-term responses can have complex effects on BEF relationships. For instance, Gravel et al.^34^,^36^ found that evolving strains of bacteria can lead to the loss of BEF relationships over evolutionary time. In contrast, Reich et al.^35^ found that BEF become steeper among plant assemblages over time.

### The effect of non-random community assembly

Several theoretical studies have documented the role of ordered extinctions in the BEF relationship^16^,^18^,^37^. These studies suggest that the order of sequential extinctions can yield concave-down relationships when species go extinct in an ordered sequence from the least efficient to the most efficient, or on the contrary, concave-up relationships when species go extinct in an ordered sequence from the most efficient to the least efficient.

**Figure 5:**
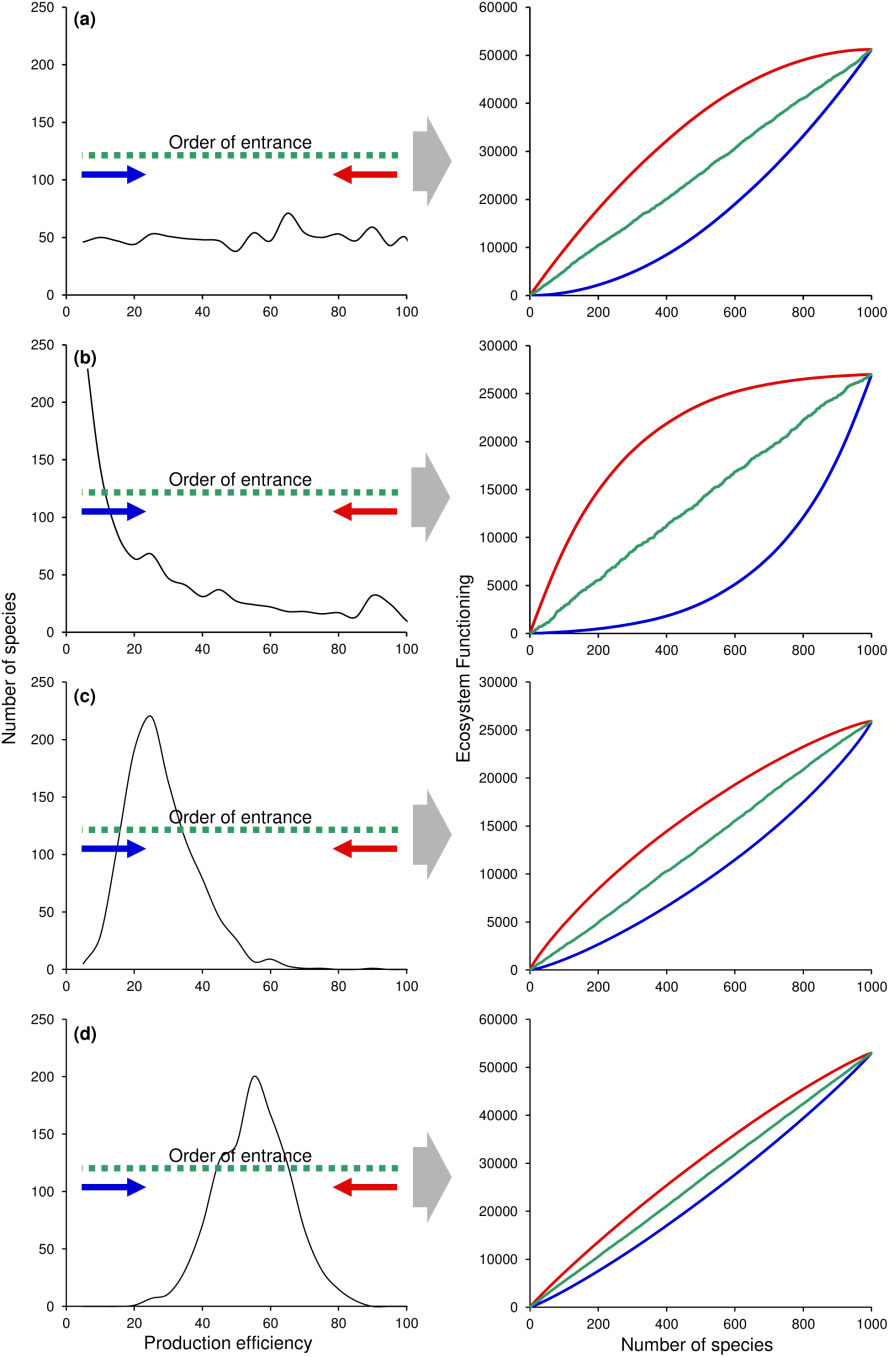
Effect of the order of community assembly on biodiversity-ecosystem functioning relationships. Plots to the left show different frequency distributions of species's production efficiency. Plots to the right show the biodiversity-ecosystem functioning relationship resulting from a community assembly process in which species are added in a given order. Blue lines indicate cases in which species are added from low to high efficiency (this is analogous to removing species from high to low efficiency). Red lines indicate cases in which species are added from high to low efficiency (this is analogous to removing species from low to high efficiency). Green lines indicate cases in which species are added at random.

An alternative interpretation for these patterns is that ecosystems are assembled in a successional order. For instance, ecosystems are likely to be colonized initially by small-bodied species (because they disperse faster or because they are more common than large-bodied species) and by lower trophic levels. Over time, colonization by larger-bodied species and higher trophic levels will occur as the presence of smaller species and lower trophic levels provides the energetic conditions for their persistence. Since species that are larger and belong to higher trophic levels tend to accumulate more biomass than smaller species from lower trophic levels, the former have a higher production efficiency. Mathematically, the ordered addition of more efficient species will tend to increase the power parameter ***1+d-c*** in equation 6 and thus increase the slope of the BEF relationship. Patterns of BEF relationships for contrasting ordered additions by body size or production efficiency are shown in Fig. 5.

It should be noted that ordered colonizations (through succession) and ordered extinctions are different mechanisms dealing with different aspects of community assembly and disassembly, respectively. Yet both of these processes cause similar effects on the shape of the BEF relationship. Ordered colonization from the least to the most efficient species yield a concave-up BEF relationship, just as do ordered extinction from the most to the least efficient species (see blue lines in Fig. 4). The prevalence of either mechanism (i.e., succession or ordered extinction) in natural ecosystems is to be determined, although they are both differentially supported for the marine observational studies considered here. Ordered colonization is likely to occur in both coral reef fishes and deep-sea nematodes whereas ordered extinctions may only apply to coral-reef fishes. Reef fishes can be differentially fished according to their body size and trophic level, while deep-sea small invertebrates (such as nematodes) occupy a relatively stable environment and thus there is little indication they will be driven to extinction preferentially by body size or trophic level given human activities.

## Concluding remarks

Our theoretical framework allows explaining and reconciling contrasting BEF relationships found in different systems based on how species are grouped in functional groups, species interactions and patterns of succession (Figs 2,3,4,5). For instance, two studies in natural temperate terrestrial plant ecosystems have found BEF relationship varying from concave-down^38^ to flat^39^. Our results also suggests that the concave-down patterns commonly found in experimental studies, in which only relatively few species are confined to artificial experimental settings, may arise as a result of: *i)* exacerbated negative ecological interactions among species, *ii)* lack of species in the assembled community that could interact positively; and/or *iii)* the short duration of most experimental studies, which are likely to mimic random or very early stages of succession. By contrast, patterns found in coral-reef fishes and in deep-sea nematodes may reflect ecological and evolutionary processes that allow niche specialization and/or mature successional communities that already contain species with a higher production efficiency. Our theoretical framework is supported by recent long-term experiments showing a steepening of the BEF relationship over time^34^,^35^, which could emerge from increased specialization.

Although we framed our study on biomass production, there is no reason to expect that the hypotheses developed here cannot be generalized to other ecosystem processes and services. First, it should be noted that biomass production is an important and broadly studied ecosystem process; and, thus our hypothesis are relevant to a broad expand of accumulated knowledge on this ecosystem process. Secondly, rare studies on single (e.g. nutrient cycling^40^) or multiple independent^12^ and simultaneous^41^,^42^ processes have shown similar BEF relationships. The similarities of patterns with other processes suggest that the hypotheses based on biomass production could be generalized to other ecosystem processes. For instance, Fründ et al.^30^ have recently showed that plant pollination by bees increases with pollinator diversity because of niche shifts that make pollinators more specialized and more efficient, in agreement with our second hypothesis. As another example, Larsen et al.^37^ showed that ordered extinctions could yield concave-up relationships between biodiversity and dung burial rate, in agreement with our third hypothesis. There are relatively other few examples with other processes we can use, which is to point out a clear need to study more functions in natural ecosystems.

In conclusion, our study provides a simple and parsimonious set of hypotheses to reconcile the contrasting BEF relationships found in experiments and some observational studies. If the hypotheses we propose are correct, they suggest that *i*) in diverse ecosystems like coral reefs and the deep-sea, the high number of species can enhance species' production efficiency through niche specialization and/or by triggering demographic and morphological adaptations that increase their biomass production, *ii*) the order of succession and/or extinctions plays a significant role in ecosystem functioning, *iii*) many species, more so than those typically included in experimental studies, are necessary to maintain the functioning of these natural ecosystems [see also^43^], *iv*) redundancy is not as pervasive in natural communities as it is in experiments (i.e., most species are specialized in natural ecosystems), and thus, the extinction of any single species may have irreplaceable ecosystem consequences, and finally, *v*) the consequences of biodiversity loss could be substantially larger in natural ecosystems than previously anticipated by experimental manipulative studies (i.e., the loss of a single species in a natural ecosystem could have exponential reductions in ecosystem functioning as oppose to the linear or saturating effect expected from experimental results). Finally, our study confirms the importance of further attempts to highlight the differences between laboratory and field, and marine vs other ecosystems. Clearly, natural ecosystems are revealing a larger complexity that that indicated by experimental setups [see also^8^] and this could have broad consequences not only on our ecological knowledge but on the implications of ongoing biodiversity loss.

Further studies are needed, however, to further explore the implications of these findings. For instance, our hypotheses suggest that it would be important to perform large-scale manipulative studies that include a larger number of species than in previous studies. Another important development would be represented by a broader and more thorough comparison of the slopes of the BEF relationships in experimental vs observational studies and in different types of ecosystems. Lastly, we suggest that future BEF studies will greatly benefit from measuring detailed individual-level responses to changes in biodiversity, in particular those responses that affect species' production efficiency, such as niche shifts as revealed in recent studies^29^,^30^.

## Author Contributions

C.M. and M.L. conceived the study. C.M. and R.D. provided the data sets. C.M., R.D. and M.L. analyzed the data. C.M. and M.L. performed the modeling. C.M., R.D. and M.L. contributed to the writing of the manuscript.

## Figures and Tables

**Table 1:** Power parameters of the relationships between biodiversity and ecosystem functioning in coral-reef fishes and deep-sea nematodes

	Power exponent (R^2^)		
	Ecosystem functioning vs		
Region	Species richness	Functional richness	Data from
**Coral reefs** (Functioning as standing stocks; g/50m^2^)			
*Caribbean*	1.8 (0.53)	3.0 (0.44)	Mora et al. 2011
*Eastern Pacific*	2.6 (0.60)	4.3 (0.40)	Mora et al. 2011
*Indo Pacific*	1.2 (0.38)	2.3 (0.38)	Mora et al. 2011
*Indian Ocean*	1.1 (0.58)	2.3 (0.50)	Mora et al. 2011
**Deep-sea** (Functioning as standing stocks; mgC/m^2^)			
*Pacific*	3.2 (0.1)	8.4 (0.21)	Danovaro et al. 2008
*Atlantic*	3.1 (0.36)	7.6 (0.20)	Danovaro et al. 2008
*Mediterranean*	1.9 (0.53)	3.2 (0.1)	Danovaro et al. 2008
